# The relationship between user interface problems of an admission, discharge and transfer module and usability features: a usability testing method

**DOI:** 10.1186/s12911-019-0893-x

**Published:** 2019-08-24

**Authors:** Razieh Farrahi, Fatemeh Rangraz Jeddi, Ehsan Nabovati, Monireh Sadeqi Jabali, Reza Khajouei

**Affiliations:** 1 Student Research Committee, Department of Health Information Management & Technology, School of Allied Health Professions, Kashan University of Medical Sciences, Kashan, Iran; 2 Health Information Management Research Center, Department of Health Information Management & Technology, School of Allied Health Professions, Kashan University of Medical Sciences, Kashan, Iran; 3 Medical Informatics Research Center, Institute for Futures Studies in Health, Kerman University of Medical Sciences, Kerman, Iran

**Keywords:** Hospital information system, Usability evaluation, User Interface, Effectiveness, Efficiency, Satisfaction

## Abstract

**Background:**

The admission, discharge and transfer (ADT) module is used in the hospital information system (HIS) for the purposes of managing appointments, patient admission, daily control of hospital beds, planning surgery procedures, keeping up-to-date on patient discharges, and registering patient transfers within or outside the hospital. The present study aimed to evaluate the usability of ADT module of a HIS through usability testing and assess the relationship between the number of user interface problems and usability features (i.e. effectiveness, efficiency, and satisfaction).

**Methods:**

This descriptive analytical study was conducted in Shahid Beheshti hospital in Kashan, Iran, in 2017. The participating users were eight students in their last semester of a Bachelor of Health Information Technology Sciences degree. First, the users were introduced to the module functions in a two-hour session; ten days later, the users were asked to perform scenarios designed based on seven tasks and take notes of the problems encountered in performing each task after it was over. Effectiveness was measured based on the rate of completing the tasks, efficiency based on the time taken to perform each task, and satisfaction based on the users’ answers to a satisfaction questionnaire. The relationship between these three usability features and the number of problems noted was assessed using Spearman’s test in SPSS version 16.

**Results:**

Thirteen unique usability problems were identified from the perspective of the users. Effectiveness was rated as 58.9%, efficiency as 53.3%, and mean user satisfaction as 53.4 ± 10.6. The number of problems in each task had significant relationships to the effectiveness (*P* = 0.009) and efficiency (*P* = 0.016) scores. User satisfaction also had a significant relationship with the effectiveness (*P* = 0.043) but not with the efficiency (*P* = 0.230) scores.

**Conclusions:**

In the view of the potential users, a HIS, used in more than 200 hospitals in a developing country, has several usability problems in its ADT module and its effectiveness, efficiency, and user satisfaction were not acceptable. The number of usability problems in the HIS user interface affected the effectiveness, efficiency and user satisfaction of the system.

## Background

Hospital information systems (HISs) are among the most popular health information systems that can increase the efficiency of healthcare providers, improve the quality of care, and increase patient safety by supporting care activities, increasing the speed and accuracy of performing tasks, and reducing errors [1–3].

Despite the many benefits of HISs, there are also some barriers related to the use of them [4]. One of the barriers is poor usability which leads to a reduced acceptance of the system, increased errors, and reduced user efficiency and can even adversely affects patient safety [5–7]. Poor usability and system failure have been observed in many HISs implemented so far [8–10]. The international standard organization (ISO) has defined the usability of systems with three features of effectiveness, efficiency and satisfaction [11], and user interface problems may be associated with these features. Studies [12, 13] have shown that usability problems such as overcrowded pages and many steps to perform a task entail reduced user productivity.

Resolving usability problems can increase user effectiveness and efficiency, such that effectiveness and efficiency improved significantly in one study [14] as a result of modification of identified usability problems. In another study, Saleem et al. [15] showed that redesigning a clinical reminder system based on the problems identified in its usability evaluation significantly improved the time taken for completing the processes (i.e. improved efficiency). In another study, Karahoca et al. [16] found that the tasks defined in emergency information system are performed significantly faster when using a user interface that has a high usability score (i.e. a higher efficiency).

The Admission, discharge and transfer (ADT) module is used in the HIS for the purposes of managing appointments, patient admission, daily control of hospital beds, planning surgery procedures, keeping up-to-date on patient discharges, and registering patient transfers within or outside the hospital [17]. Several studies have identified the usability problems of this module [18–20]. A better insight into the usability of this module requires not only the identification of its usability problems, but also determination of the relationship between these problems and usability features, i.e. effectiveness, efficiency and satisfaction [21, 22]. No studies were found that both identified the usability problems of this module and investigated the relationship between number of problems and usability features. The present study was conducted to identify the usability problems of the ADT module in a HIS by usability testing from the perspective of health information technology students as potential system users. The relationship between the number of identified problems and usability features was then also assessed.

## Methods

The usability of the ADT module was assessed through usability testing [23] and the usability problems were recorded by potential users. Assessing the usability problems with this method often leads to the identification of 80–85% of the problems by five to eight users [24–26].

### Study population and setting

The participants were eight senior bachelor students of Health Information Technology Sciences who volunteered to take part. The study inclusion criteria were: (1) familiarity with health information management department tasks; (2) computer skills; and (3) no working experience with the ADT module.

This study was conducted in the health information management department of Shahid Beheshti hospital, affiliated to Kashan University of Medical Sciences, in Kashan, Iran, in summer 2017. This hospital is the largest one affiliated with this university and has 615 registered beds.

### Description of the evaluated hospital information system

Shafa HIS is developed and supported by Tirajeh Rayaneh Co. and is an information system that organizes key hospital management and clinical activities. This system covers all hospital activities, from patient admission to discharge, based on a patient-oriented approach, and stores all the data in the patient’s electronic health record. At the time of performing this study, more than 200 hospitals in Iran were covered by the services of this company [27].

### Assessed tasks

In a session with the participation of the manager of health information management department and after assessing the health information management department tasks, seven main and common tasks performed through the ADT module were selected (Table 1).

**Table 1:** Main and common tasks performed through the ADT module

Number	Task
1	Inpatient admission
2	Outpatient admission
3	Reporting of the services provided to the patient
4	Reporting of the hospital departments performance
5	Recording the diagnostic codes
6	Reporting of disease diagnostic codes
7	Editing patient data

Scenarios were designed based on these tasks and using real data while preserving the patient data confidentiality. These scenarios were modified and approved by the manager of the health information management department.

### Evaluation method

This study used the usability testing method to identify the system problems, and three features (i.e. effectiveness, efficiency and user satisfaction) were measured. Usability testing is a user-based usability evaluation method [23]. In this study, the users performed their tasks through the information system and, at the same time, the evaluator took notes of the problems they encountered during interaction with the system.

### ISO-based usability features [11]

#### Effectiveness

The extent to which the user can fully and accurately achieve his goals in performing a task [28–31]. Effectiveness was measured using the following equation [32].

$$ \mathrm{Effectiveness}=\left(\left(\mathrm{number}\ \mathrm{of}\ \mathrm{successfully}\ \mathrm{completed}\ \mathrm{tasks}\right)/\left(\mathrm{total}\ \mathrm{number}\ \mathrm{of}\ \mathrm{tasks}\ \mathrm{performed}\right)\right)\ast 100 $$

The range of effectiveness was taken as ‘awful’ (0–50%), ‘bad’ (50–75%), ‘normal’ (75–90%), and ‘good’ (90–100%) [33].

#### Efficiency

The mean time taken for the users to perform each task [28, 34, 35] based on the following equation [32]:

$$ \mathrm{Efficiency}=\left(\left(\mathrm{totaloffullcompletionofatask}(1)\mathrm{ornon}-\mathrm{completion}(0)/\mathrm{timespentonatask}\right)/\left(\mathrm{totalnumberoftasks}\ast \mathrm{numberofusers}\right)\right)\ast 100 $$

In the above formula, the value of one means that the task has been fully performed by the user and the value of zero means that the task has not been fully performed. This value is divided by the time spent on a task. The calculation is done for all the tasks performed by users and the values are added up. Finally, the result is divided by the multiplication of the total number of tasks and the number of users to calculate system efficiency in percentage.

#### Satisfaction

Participants’ satisfaction was assessed using the System Usability Scale (SUS) [36]. The validity and reliability of the Persian version of this scale had been confirmed in a study conducted by Taheri et al. [37]. This scale measures “the perceived ease of use” of a system. It contains ten items on a five-point Likert scale, and its scores are calculated based on Brooke’s scoring guideline [36]. According to previous studies [28, 38, 39], a mean score ≤ 50 is taken as poor satisfaction (i.e. ‘not acceptable’), a score between 50 and 70 taken as a system requiring modification but deemed ‘passable’, between the high 70 and 80 were considered as ‘good’, and a score 90 and above were deemed as ‘superior’.

### Data collection

The participants were first introduced to the ADT module functions in a two-hour session. To prevent any learning effect, after a washout period of 10 days, they performed the tasks based on the designed scenarios. They were asked to take note of the usability problems encountered in each task and the reasons for completing or not completing the tasks. One researcher supervised the evaluation session, but neither user received instruction during the task performance stage. The users completed the user satisfaction questionnaire afterwards. Effectiveness was measured based on the ‘completion’ or ‘non-completion’ of the tasks. The users were asked to announce the time of completion of each task or the time when they were no longer able to complete the task. The efficiency feature was measured by recording the time taken to perform each task.

### Data analysis

Data were analyzed in Excel 2013 using descriptive statistics, and the relationship between the three usability features and the number of usability problems was assessed in SPSS 16.0 (SPSS Inc., Chicago, IL, USA) using Spearman’s test at the significance level of 0.05.

## Results

Of the 56 tasks performed by the eight users (seven tasks per user), 33 (59%) were completed and 23 (41%) were left incomplete. Totally, 36 usability problems were identified in this evaluation, but 13 remained after the elimination of duplicates cases. Table 2 shows the problems expressed by the users and their related tasks.

**Table 2:** The problems identified by the users in usability testing

Number	Problem	Number of users expressing the problem	Task number
1	The format of some components of the user interface, such as the icons, is ambiguous and the signs do not imply their function.	5	All tasks
2	Performing some tasks through different parts of the system is problematic for the user (such as generating statistical reports).	4	3, 4
3	The menus automatically hide on the side of the page, making user access to them difficult.	4	All tasks
4	Some icons are disabled while they have to be enabled.	4	5
5	The heading to filter the reports is not detectable by the users	3	6
6	The data of each section is not mentioned in its subsets in a cohesive manner.	3	4
7	The items do not have clear and proper labels.	3	3, 4
8	Some buttons are not visible for the users.	2	7
9	The diagnostic report generation heading is separate from the diagnostic code recording site, which confuses the users when taking reports.	2	6
10	There are no instructions to perform a step when needed by the user.	2	All tasks
11	The small font is problematic for the users when selecting the type of diagnosis.	2	5
12	Alerts for not recorded data that must have been recorded are presented late.	1	3, 4, 5
13	To record the data, it has to be typed or its code be remembered, with no possibility of choosing between some options.	1	1, 3, 4, 5

### Effectiveness

**Fig. 1:**
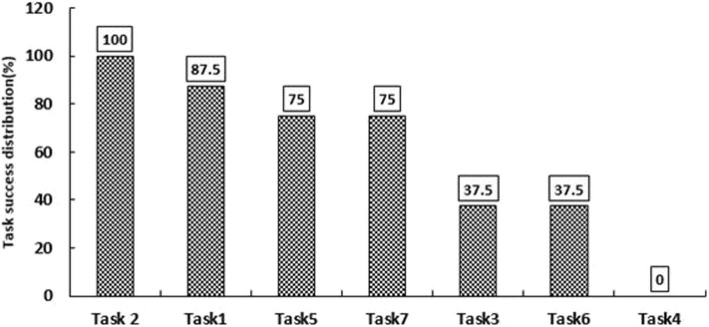
The tasks success rate

Figure 1 presents the results on the tasks’ effectiveness. Task 2 (i.e. outpatient admission) was performed by all the users. The users reported the simplicity, not needing to know the next steps of the task and existence of data recording prompts as the reasons for performing this task in full. Task 4 (i.e. reporting on the performance of the hospital departments) was not performed completely by any of the users. The reasons given by the users for not completing this task were having to perform the task in two different parts of the system, the unclear function of the items on the page, the many steps of the task, and the lack of help when performing the task.

According to the results, the effectiveness of the ADT module was 58.9%. Spearman’s correlation test showed a significant, inverse, linear relationship between effectiveness and the number of problems in each task (*P* = 0.009, r = − 0.881). Spearman’s test also showed a significant relationship between the effectiveness and efficiency scores of each user (*P* = 0.039, r = 0.731).

### Efficiency

Table 3 presents the mean time taken to perform the tasks. Task 7 (i.e. editing the patient data) took the shortest time. Simplicity, single-step nature of the task, and no need to complete some data elements were the reasons given by the users for the short time taken to perform this task. Task 1 (i.e. inpatient admission) took the longest time. The large number of data elements in the admission form and the fact that the elements were on two separate pages were the reasons given by the users for the long length of time taken to complete this task.

**Table 3:** Mean time to perform the tasks

Task		Task 7	Task 6	Task 2	Task 3	Task 5	Task 4	Task 1
Time per task (minute)	Mean (SD)	0.82 (0.62)	1.74 (1.10)	1.84 (0.61)	1.98 (0.82)	2.20 (0.57)	2.80 (1.12)	5.18 (1.02)
	Range	00.23–2.00	00.45–3.44	1.31–3.23	00.59–3.20	1.03–3.23	1.49–4.58	4.25–7.40

According to the noted equation, the system’s relative overall efficiency was 53.3%. Spearman’s correlation test showed a significant, inverse, linear relationship between efficiency and the number of usability problems in each task (P = 0.016, r = −0.847).

### Users’ satisfaction

**Fig. 2:**
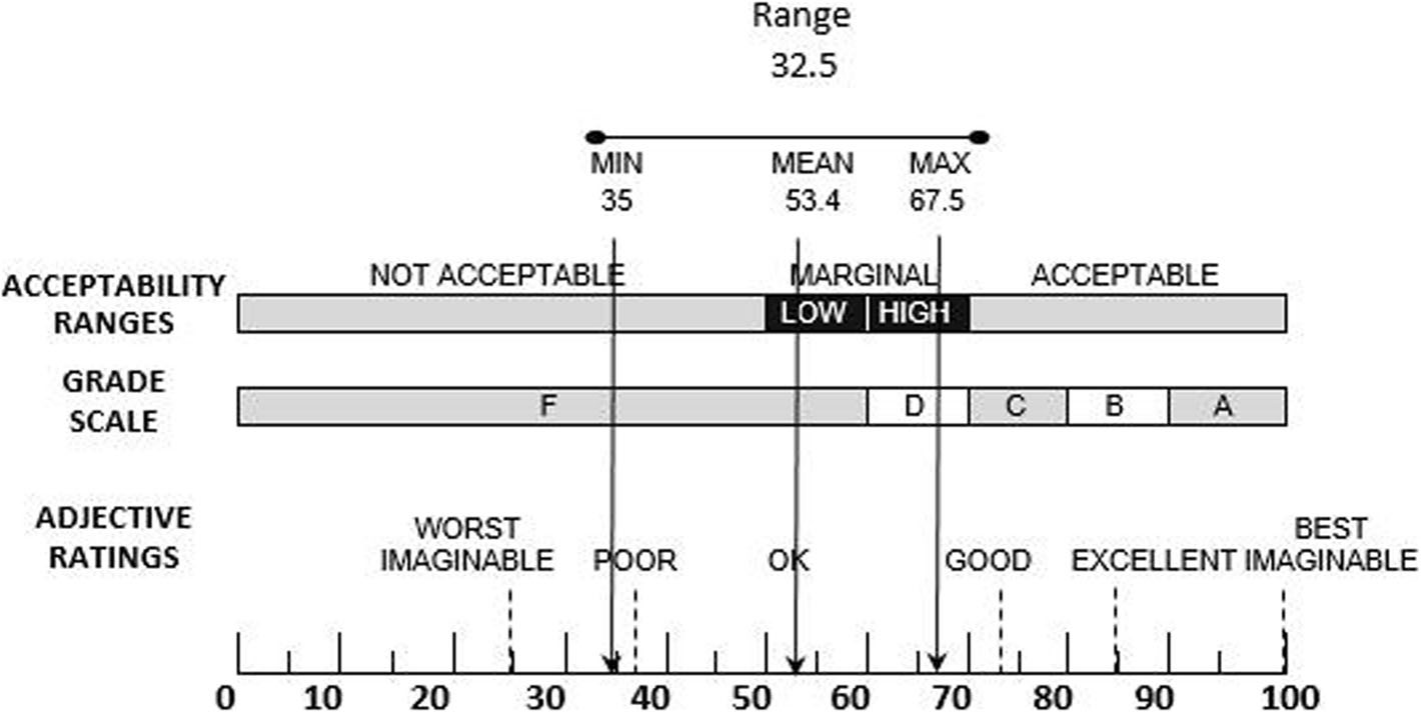
Overview of modified SUS rating table [38]

The mean user satisfaction score was 53.4 ± 10.6, and according to Fig. 2, satisfaction with the system was at a borderline acceptability with grade F. Spearman’s test results showed a significant relationship between user satisfaction and the task effectiveness score (*P* = 0.043, r = 0.722). No significant relationships were observed between user satisfaction and the efficiency score (*P* = 0.230, r = 0.479).

## Discussion

In this study, 13 unique usability problems were identified by the potential users for the ADT module. Effectiveness was < 60%, Efficiency about 50%, and mean users’ satisfaction about 50. Significant inverse relationships were found between the effectiveness score and the number of problems and also between the efficiency score and the number of problems in each task. Significant relationships were also found between the effectiveness score and users’ satisfaction and also between the effectiveness score and the efficiency score of users.

Out of the 13 unique usability problems, three were common to all the tasks: (1) an automatically-hiding menu bar and it’s unclear retrieval icon; (2) the unclear function of the keys based on their icon; and (3) the absence of help. The users argued that the first problem made accessing the other parts of the system troublesome. Similarly, Li et al. [39] argued that the lack of navigation control was an important problem that made the users’ access to the other parts of the system difficult. In the present study, the users reported that the second and third problems were confusing and wasted their time. The results of other usability studies [40, 41] using expert-based usability evaluation methods confirm the presence of these two problems in other HISs. Health information system designers and developers should therefore consider the modification of the poor navigation control in HISs as high priority, which exists from the joint perspective of experts and users.

In the present study, less than 60% of the tasks were completed, which shows the poor effectiveness of the system. The results also showed that the number of usability problems had an inverse relationship with effectiveness. Similarly, in their study, Thyvalikakath et al. [42] argued that there is a strong relationship between the frequency of usability problems in a computer-based patient record system and the users’ failure to perform the tasks. None of the users in our study were able to fully perform one task (i.e. “reporting on the performance of the hospital departments”), which had the largest number of problems compared to the others. The reasons given by the users for not performing this task included performing one task in two different parts of the system, the unclear function of the items in the page, the multiple steps of the task, and the absence of help when performing the task. Unlike the noted task, another task (“outpatient admission”) was performed in full by all the users and had the smallest number of problems compared to the other tasks. The reasons given by the users for fully completing this task included simplicity, not requiring to know the next steps and data recording prompts for the users.

The efficiency of the evaluated system was about 50%, which is considered poor. The “inpatient admission” task took the longest time. The large number of data elements in the admission form and their placement on two separate pages had prolonged this task according to the users. Moreover, “editing the patient data” task took the shortest time, which, according to the users, was due to the task’s simplicity, single-step nature, and not needing to complete some the data elements. The present findings also showed that greater usability problems decrease efficiency. In another study [43], user interface problems affected the nurses’ efficiency in using an electronic medication administration record application. With a larger number of usability problems in user interface, the user has to spend more time to perform a task, and his efficiency of performing the task therefore reduces. The results also showed a significant relationship between the efficiency and effectiveness scores of performing the tasks in each user. Similarly, Georgsson et al. [28] showed that users who successfully completed their tasks (i.e. greater effectiveness) performed their tasks more quickly (i.e. with higher efficiency).

The present findings showed that the overall user satisfaction of the system was low, and there was a significant relationship between the satisfaction and effectiveness scores. For example, the “edit” icon for editing the patient record number was poorly located on the page, and finding it required a long time or led to the unsuccessful task completion. The researcher observed up close that this problem had led to user dissatisfaction. The results of another study [31] showed that user satisfaction increases when the system is efficient and effective and the users can better perform their tasks. Moreover, the results of other studies [44, 45] showed that user satisfaction with the CPOE system is strongly tied to the system’s ease of use and its response efficiency and time.

The present study was conducted with the participation of potential users in a real setting and using actual scenarios. To the researchers’ knowledge, students have not yet been used as potential users for assessing HISs to identify their usability problems. The present study first identified the usability problems and measured three usability features (i.e. effectiveness, efficiency and satisfaction) and then assessed the relationship between the number of problems and the usability features. This study also had some limitations. Due to the homogeneity of the study population, the analysis of the results based on the users’ demographic features was not possible. The small number of the users could have affected the statistical generalizability of the results. Also, due to not including all the tasks performing through the ADT module, the users could not have carried out a systematic search of the system problems; therefore, the ADT module should have other problems that have remained unidentified.

The results revealed the participants’ dissatisfaction with the ADT module and its inadequate effectiveness and efficiency. The researchers recommend that certain parts of the system that showed a large number of problems be redesigned. A larger-scale study is recommended to be conducted with a larger number of users randomly selected to enable the comparison of the users’ demographic features and functional parameters such as the rate of completion of tasks and the time taken to perform the tasks.

## Conclusion

In view of the potential users, the examined HIS, which is used in more than 200 hospitals in a developing country, has many usability problems in terms of its user interface in the ADT module. Moreover, the system effectiveness and efficiency and the users’ satisfaction were not at an acceptable level. The number of problems identified in the HIS user interface affected the effectiveness and efficiency of the system and the users’ satisfaction. To improve these features before and while using the system, these usability problems should be resolved.

## Data Availability

The data generated and analyzed during this study are available from the corresponding author on reasonable request.

## Abbreviations

ADT: Admission, discharge and transfer
CPOE: Computerized physician order entry
HIS: Hospital information system
ISO: International standard organization
SUS: System usability scale
